# Expression of Ovine Herpesvirus -2 Encoded MicroRNAs in an Immortalised Bovine – Cell Line

**DOI:** 10.1371/journal.pone.0097765

**Published:** 2014-05-21

**Authors:** Katie Nightingale, Claire S. Levy, John Hopkins, Finn Grey, Suzanne Esper, Robert G. Dalziel

**Affiliations:** 1 The Roslin Institute & R(D)SVS, University of Edinburgh, Edinburgh, Midlothian, United Kingdom; University of Liverpool, United Kingdom

## Abstract

Ovine herpesvirus-2 (OvHV-2) infects most sheep, where it establishes an asymptomatic, latent infection. Infection of susceptible hosts e.g. cattle and deer results in malignant catarrhal fever, a fatal lymphoproliferative disease characterised by uncontrolled lymphocyte proliferation and non MHC restricted cytotoxicity. The same cell populations are infected in both cattle and sheep but only in cattle does virus infection cause dysregulation of cell function leading to disease. The mechanism by which OvHV-2 induces this uncontrolled proliferation is unknown. A number of herpesviruses have been shown to encode microRNAs (miRNAs) that have roles in control of both viral and cellular gene expression. We hypothesised that OvHV-2 encodes miRNAs and that these play a role in pathogenesis. Analysis of massively parallel sequencing data from an OvHV-2 persistently-infected bovine lymphoid cell line (BJ1035) identified forty-five possible virus-encoded miRNAs. We previously confirmed the expression of eight OvHV-2 miRNAs by northern hybridization. In this study we used RT-PCR to confirm the expression of an additional twenty-seven OvHV-2-encoded miRNAs. All thirty-five OvHV-2 miRNAs are expressed from the same virus genome strand and the majority (30) are encoded in an approximately 9 kb region that contains no predicted virus open reading frames. Future identification of the cellular and virus targets of these miRNAs will inform our understanding of MCF pathogenesis.

## Introduction

Malignant catarrhal fever (MCF) is a fatal disease of cattle, deer and pigs caused by one of two related gammaherpesviruses (γ-HV), ovine herpesvirus -2 (OvHV-2) or alcelaphine herpesvirus 1 (AlHV-1). MCF is characterized by sudden onset of fever followed by lymphadenopathy, leukocytosis, severe congestion, necrosis and erosion of the oral, conjuctival and nasal mucosæ [1]. The disease occurs as the result of infection of susceptible hosts by contact with an asymptomatic carrier species that acts as a virus reservoir. OvHV-2 is the major cause of sheep associated MCF worldwide [1]. It infects most sheep, where it establishes a latent but asymptomatic infection. AlHV-1 is the major cause of MCF in sub-Saharan Africa where the wildebeest is the asymptomatic carrier species.

The same cell populations are infected in both cattle and sheep [2], [3] but only in cattle does virus infection cause dysregulation of cell function leading to uncontrolled proliferation, cytotoxicity and disease. In both species the infected cells arise from common lymphoid progenitors [4] but interleukin 2-dependent cell lines can be cultured only from affected animals. There is currently no tissue culture system for OvHV-2 and such cell lines are the only resource for working with the virus. In affected species the infected cells have been described as large granular lymphocytes (LGLs) [5]. The immortalized cell lines have a variety of phenotypes; all express CD2 but vary in their expression of CD4 and CD8 [2], [3]. The mechanism by which OvHV-2 induces MCF is unknown; virus-induced cytopathology is thought not to be involved in lesion development and it has been proposed that tissue damage arises from non-antigen specific, MHC unrestricted cytotoxicity of the LGLs. The key question in understanding OvHV-2 pathogenesis is; why infection of the same cell type in two closely related species leads to such different disease outcomes, i.e. lymphoid cell dysregulation and MCF in cattle versus asymptomatic infection in sheep.

MicroRNAs (miRNAs) constitute a large family of small, non-coding RNAs functioning in post-transcriptional regulation of mRNA in eukaryotes [6]–[8] as well as in a number of viruses, particularly in the members of the family *Herpesviridae* [9]–[11]. miRNA regulation of expression is by binding of the miRNA seed sequence (∼nucleotides 2–8) to complementary sequences in target mRNAs and directing these for degradation or translational silencing; the majority of miRNAs target sequences within the 3′ UTR [6]. Herpesvirus-encoded miRNAs have been shown to be effective regulators of both cellular and virus gene expression and to influence cell processes including the cell cycle [12]–[17]. The pathology of the herpesviruses Epstein Barr virus (EBV) and Marek's disease virus (MDV) also involves aberrant lymphocyte proliferation, and virus-encoded miRNAs play a key role in the induction of this proliferation. Furthermore, deletion of a single (MDV) or a small cluster (EBV) of virus-encoded miRNAs attenuates these viruses [18]–[20]. Consequently we hypothesized that OvHV-2 encodes miRNAs that target host mRNAs and that these constrain virus pathology in sheep and/or induce MCF pathology in cattle. Identification and characterization of OvHV-2-encoded miRNAs is therefore essential to allow this hypothesis to be addressed.

In a previous study we reported the results of massively parallel sequencing of small RNAs present in the BJ1035 OvHV-2 immortalized bovine LGL cell line and predicted that the virus encodes up to forty-five miRNAs [21]. Eight of these were confirmed by northern hybridization [22]. Here we extend these studies using two PCR protocols to investigate the expression of the other predicted OvHV-2 miRNAs.

## Methods

### Cell culture

BJ1035 cells, an immortalized bovine T cell line from an animal naturally infected with OvHV-2 [3], was grown in suspension culture in Iscove's Modified Dulbecco's Medium (Invitrogen, Paisley, UK) supplemented with 10% (v/v) foetal calf serum (Sera Laboratories International, Haywards Heath, UK), 1% (v/v) penicillin-streptomycin (Invitrogen), 20 U/ml interleukin 2 (Novartis Pharmaceuticals UK, Camberley, UK) and incubated at 37°C, 5% CO_2_. Bovine lymphoid cells were isolated from fresh blood and lymphoblasts generated as described previously [23]. All relevant procedures were approved by the University of Edinburgh Ethical Review Committee and carried out under an Animal (Scientific Procedures) Act 1996 project licence.

### Reverse Transcription PCR (RT-PCR)

Bovine lymphoblasts and BJ1035 cells were pelleted by centrifugation and total RNA extracted using Trizol (Invitrogen). cDNA was generated using the miScript II RT Kit (Qiagen, Crawley, UK) that uses oligo-dT to prime reverse transcription. For each predicted miRNA, a specific forward primer spanning the first 15–16 nucleotides of the miRNA was designed (Table S1) allowing subsequent sequence confirmation of the identity of the amplified miRNA RT-PCR was performed using miScript SYBR green PCR kit (Qiagen), with the supplied universal reverse primer and the miRNA specific forward primers using the annealing temperatures shown in Table S1. PCR products were fractionated by electrophoresis using 3% agarose, purified using the Illustra GFX PCR DNA and Gel Band Purification Kits (GE Healthcare, Amersham, UK) and cloned using the TOPO TA Cloning Kit (Invitrogen). Plasmid DNA was isolated using the Qiaprep Spin Miniprep Kit (Qiagen), analysed by restriction enzyme digestion and sequenced (GATC Biotech, London UK).

### miRNA specific reverse transcription

Individual miRNA specific primers (Table S2) were used to prime for reverse transcriptase. Each primer also has a 5′ conserved region recognised by a universal reverse primer (5′- GTGCAGGGTCCGAGGT-3′) [24]. An initial reaction to form the hairpin structure of the reverse transcription primer (200 µM) was carried out using the following conditions: 95°C for 30 min, 72°C for 2 min, 37°C for 2 min then 25°C for 2 min. The concentration of the reverse transcription primer after annealing was 50 µM. For the reverse transcription reaction 5 µM reverse transcription primer was used to prime 100 ng of RNA. Conditions for the reverse transcription reaction were as follows: 16°C for 30 min, 42°C for 30 min then 85°C for 5 min. PCR was thenperformed using miRNA specific forward primers (Table S2) and the following conditions: 5 min at 95°C, 40 cycles of: 30 s at 95°C, 30 s at 60°C, 60 s at 72°C; then finally 7 min at 72°C. PCR products were fractionated by electrophoresis using 3% agarose. Reactions were performed in triplicate. The presence of a product in the BJ1035 cells but not in the uninfected bovine lymphoblast cells was taken as proof of OvHV-2 miRNA expression.

## Results

Using northern hybridization we have previously demonstrated the expression of the eight of the 45 predicted miRNAs that were represented by the highest number of reads in our parallel sequencing data [22]. RT-PCR using miRNA specific forward primers and a universal reverse primer for the remaining 37 predicted miRNAs initially identified 22 virus-encoded miRNAs (Table S1, Table 1).

**Table 1:** OvHV-2-encoded microRNAs.

ovhv2-miR-	Previous name	Abundance RNA-seq	Walz *et al*.	5′nt	3′nt	Sequence	Validation Method
Ov2-2	miR-1	10588	√	927	906	AAGGCUUGAUAAGUAGCACUGA	Levy *et al.*
Ov2-1		253	√	1182	1161	AUGCUUGUUUAGGCCCCAUGAA	Sequencing
17-30		434	√	27722	27702	UUUGGGUGUCUCCUGUCAUCU	Sequencing
17-29	miR-2	39169	√	27903	27881	AUCUUGGACGCAUCUGUCAGUAG	Levy *et al.*
17-28		6200	√	28066	28045	UCUAGGUUGCAUUUUGCUGUAG	Sequencing
17-27		6015	√	28209	28188	CCCACAUUUAAGGUGCUCGUGU	Sequencing
17-26		322	√	28381	28361	AUAUUCGUUUAGACGCAAGUA	Sequencing
17-25		4717	√	28515	28495	CAAUGCUGCUUUGGUGCCUCA	Sequencing
17-24		1014	√	28683	28661	GGGUUCCUCGAGUGGAUAUUGUU	Sequencing
17-23		21686	√	28781	28761	AUACACACUGAAAGAGCUAGA	Sequencing
17-22		740	√	28888	28866	AUAAGGCCAACACUAGGUGCUGU	Sequencing
17-21		31227	√	29052	29028	AAGCACCUUGGGUGAUGUCUCUGUU	Sequencing
17-20	miR-3	14694	√	29248	29226	UCUGUAUCAUAGGGGUUGUGUUG	Levy *et al.*
17-19		6487	√	29345	29323	AAGCAUAGCUGGGAGUGUCUAGA	Specific cDNA
17-18		9219	√	29455	29434	UAGUAGUCCGUUAACGCAAAGU	Sequencing
17-17	miR-4	17508	√	29637	29616	AAGGAUCCUUAAGUGACGAACG	Levy *et al-*
17-16		8095	√	29745	29725	UAAACUGGUGGUAGGCGGUCU	Sequencing
17-15		2967	√	29945	29923	UAGCAGUUAUGCAGGUAUCUGGU	Sequencing
17-14		3794	√	30091	30067	UGGCAUUUCCAGGAGCCUGUUGUUC	Sequencing
17-13		24497	√	30338	30316	UUGGGUCCAACAUGAGACGCGGU	Sequencing
17-12		14966	√	30510	30489	UAUGUCAGAAGUGAAGCUGAGA	Specific cDNA
17-11		1357	√	30632	30611	UGGUUUGCAUCUGCACCCAGUU	Sequencing
17-10	miR-5	11187	√	30818	30796	UGAAGUUACAGCUGCACCUGGAU	Levy *et al.*
17-9		5457	√	30980	30956	UAGAGUUACUAAGGAUUCCCUGGUA	Sequencing
17-8		942	√	31085	31066	AAUCGCCGGUGGCCUUCUAG	Sequencing
17-7		351	√	31359	31340	UAUAGACGGGUAUGCUGCCG	Specific cDNA
17-6	miR-6	10378	√	31462	31440	UAUUUUUAGCGGAGACCUCUAGG	Levy *et al.*
17-5		1535	√	31603	31582	CCUUUUUGGUGAGUUGCCCUGU	Sequencing
17-4		517	√	32075	32054	GAUUUGAUAAAGCCUGCCUGCG	Sequencing
17-3	miR-7	16574	√	36313	36289	GAAGGCGCAUCAUAGACACCACUUC	Levy *et al.*
17-2		7834	√	36462	36441	ACCCCGGGGGUAUGUGCAGGAC	Sequencing
17-1	miR-8	46048	√	36575	36554	UGGCUCAGCGUGACUGCUCUUC	Levy *et al.*
24-1		1	x	48585	48560	GAGCAGUACUACACAGCAGACAACAU	Sequencing
61-1		1	x	96435	96411	UUGGGGACGUGCUGGCUGACGACGU	Specific cDNA
73-1		6064	x	117122	117100	UAAUCUCUGCUCCAAUUGUAAAU	Specific cDNA

The 35 validated miRNAs are listed. **Previous name**: designation in Levy *et al.* 2012. **Abundance (RNA-seq)**: Total number of sequence tags representing the miRNA in the RNA-seq data [22]. **Walz** ***et al*** **.**: miRNAs predicted by Walz *et al*. √ = yes; X = no. **5′nt/3′nt**: the first and last nucleotides of the mature miRNA on the OvHV-2 genome (AY839756) [28]. **Sequence**: sequence of the mature miRNA. **Validation method**: Levy *et al*.: validated previously [22]; Sequencing: validated using the RT-PCR and subsequent sequencing; Specific cDNA: validated using the approach of Varkonyi-Gasic *et al*.[24].

**Figure 1:**
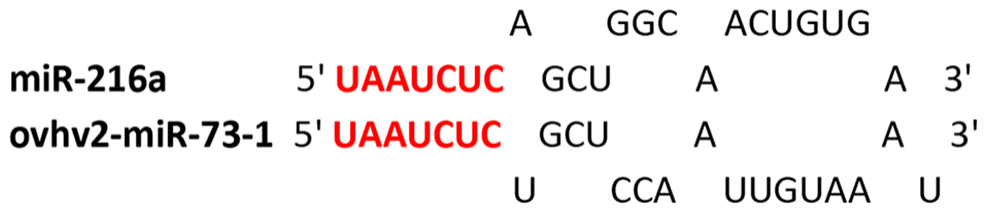
Comparison of the sequences of ovhv2-miR-73-1 and miR-216a. Seed sequences (nt 1-7) are shown in red.

**Figure 2:**
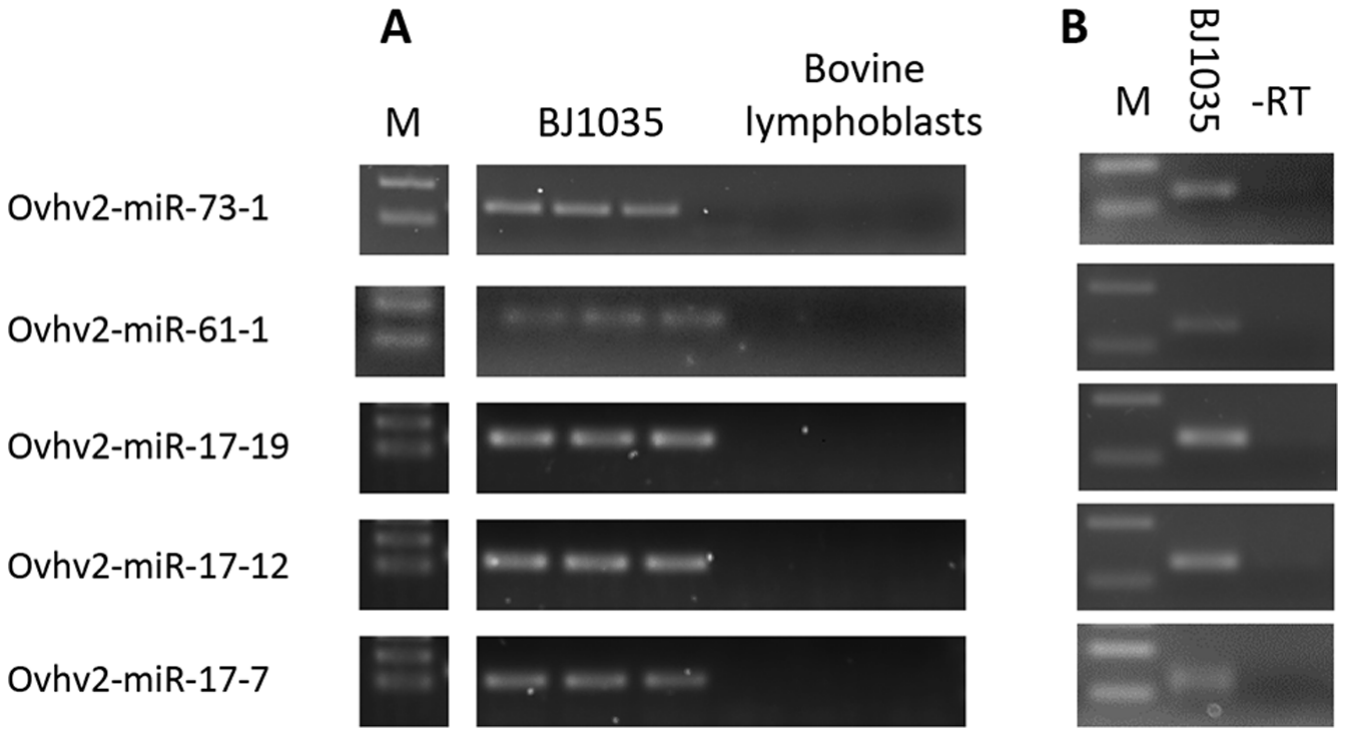
Analysis of OvHV-2-encoded miRNA expression using miRNA specific RT-PCR. A) Expression of five predicted OvHV-2-encoded miRNAs in BJ1035 cells but not uninfected bovine lymphoblasts was confirmed by miRNA specific RT-PCR. Each assay was carried out in triplicate. M: 50 bp and 100 bp markers B) miRNA specific RT-PCR was carried out along with an no RT (-RT) control.

**Figure 3:**
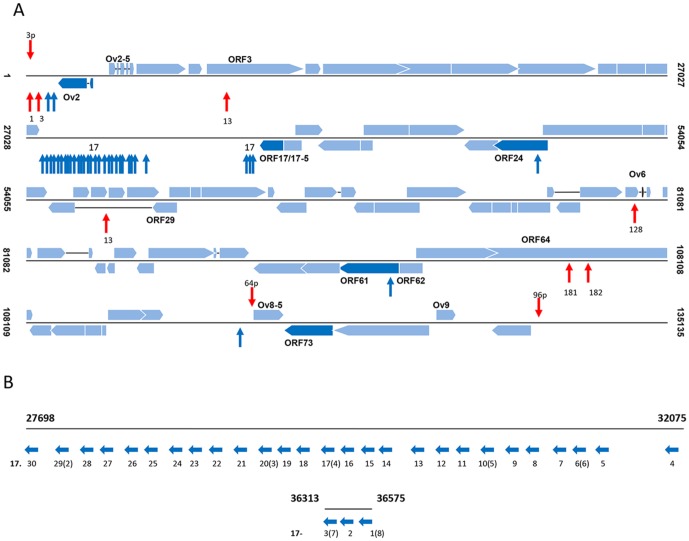
Location of miRNAs in the OvHV-2 genome. A) The relative positions of the predicted miRNAs in the OvHV-2 genome are shown. Numbering is from Hart *et al*.[28]. The genome is represented by a thin line and open reading frames (ORFs) are indicated by blue boxes. Boxes above the line represent ORFs transcribed left to right; those below the line represent ORFs which are transcribed right to left. Only those ORFs adjacent to predicted miRNAs are named. Arrows indicate the position of the predicted miRNAs; arrows above the line represent miRNAs transcribed left to right, those below the line represent miRNAs which are transcribed right to left. Dark blue vertical arrows indicate validated miRNAs. Red arrows indicate non-validated miRNAs named according to the groups listed in Table 1. Those OvHV-2 ORFs closest to validated miRNAs and after which those miRNAs are named are shown in darkblue. B) The locations of ovhv2-miR-17-1 to -30 are shown in more detail.

To investigate if the remaining 15 miRNAs are expressed, an alternative miRNA specific reverse transcription PCR strategy was adopted. The expression of a predicted miRNA (ovhv2-miR-73-1) that was shown to have seed sequence homology but no 3′ sequence homology with mammalian miR-216a (Figure 1) was also analysed by this method. The presence of a product in the BJ1035 cells but not in the uninfected bovine lymphoblast cells was taken as proof of OvHV-2 miRNA expression. As the forward primer ends at the nucleotide adjacent to that from which cDNA is primed, sequencing of the product would generate only primer sequence. To confirm that amplification was from miRNA and not genomic DNA a no RT control was performed and no amplification was observed with any primer set. Expression of a further 5 miRNAs, including ovhv2-miR-73-1, was confirmed, (Figure 2). Figure 3 shows the location and direction of transcription of the thirty-five validated OvHV-2-encoded miRNAs.

## Discussion

The number of miRNAs expressed by individual herpesviruses ranges from 8 to 50 [25] and here we demonstrate that OvHV-2 encodes 35 miRNAs. Using a predictive algorithm Walz *et al*. [26] predicted 61 hairpin sites in OvHV-2 that might encode miRNAs; 32 of which we have now confirmed to be expressed (Figure 3, Table 1), three of our validated miRNAs were not predicted by Walz *et al*.. All of the validated miRNAs expressed by OvHV-2 are transcribed in the same orientation, right to left. By convention virus-encoded miRNAs are named in relation to the nearest open reading frame (ORF) transcribed in the same direction as the miRNA; we have followed this convention in naming the OvHV-2-encoded miRNAs. Those identified in Levy *et al.* and discussed in Riaz *et al.* [22], [27] have been renamed to adhere to this nomenclature (Table 1). Two miRNAs are encoded at the left hand end of the genome in the region between the terminal repeat and the 3′ end of ORF Ov2, these have been named ovhv2-miR-Ov2-1 and ovhv2-miR-Ov2-2 (ovhv2-miR-1 in Levy *et al.* 2012[22]). The OvHV-2 genome, like that of AlHV-1 and equine herpesvirus 2 (EHV-2) contains two large regions of the genome with no predicted open reading frames [28]. The majority (30) of the identified miRNAs are encoded as two clusters at either end of the larger of these two regions. One cluster, spanning 4377 bp at the left end of this region, contains 27 miRNAs; a smaller cluster of three miRNAs is encoded in a 320 bp region at the right hand end of this region (Figure 3). These miRNAs have been named ovhv2-miR-17-1 to -30. Ovhv2-mir-17- 29, -20, -17, -10, -6, -3 and -1 were previously designated ovhv2-miR-2 to -8. One miRNA is encoded in the non-coding region situated toward the right end of the genome and is designated ovhv2-miR-73-1. The remaining miRNAs are encoded within ORFs 24 and 61 and are designated ovhv2-miR-24-1 and ovhv2-miR-61-1.

ovhv2-miR-73-1 was the only OvHV-2-encoded miRNA to show seed sequence homology to any other reported miRNA, miR-216a (Figure 1). This miRNA is broadly conserved in vertebrates and targets *PTEN* and *YBX1* [29], [30] down regulation of which can lead to increased cell survival, hypertrophy, sclerosis, and a decreased cellular response to stress [31], all symptoms observed in MCF. We are currently investigating the cellular genes targeted by ovhv2-miR-73-1.

AlHV-1 is the causative agent of wildebeest-associated MCF and the sequence identity between the individual ORFs of OvHV-2 and AlHV-1 varies from 22–83% ([28]). No significant sequence similarity was found between the non-coding regions of the two viruses and Blastn analysis failed to identify miRNAs in the AlHV-1 genome with sequence homology to any OvHV-2 miRNA. A lack of conservation of miRNAs between closely-related viruses has also been observed in different Marek's disease virus strains [32].

Herpesviruses are generally host species specific and are considered to have co-evolved with their natural host [33]. Herpesvirus-encoded miRNAs have been shown to regulate both virus and host gene expression [12]–[17] and it is likely that these miRNAs have also co-evolved with their host targets. The different disease outcomes seen in sheep (natural, co-evolved host) and cattle (“foreign” host may be the result of different virus:host interactions. Our hypothesis is that OvHV-2-miRNAs interact differently with sheep and cattle genes and that these differences play a role in the differing disease outcomes. It is likely that AlHV-1 does encode miRNAs, but that they have co-evolved to target wildebeest (natural host) genes in a similar manner to which OvHV-2 miRNAs have evolved to target sheep genes.

The identification of the miRNAs encoded by OvHV-2 allows us to study how these miRNAs affect host and virus gene expression. We have recently shown that viral genes ORF20 (cell cycle inhibition), ORF 50 (reactivation) and ORF 73 (latency maintenance) are targeted by ovhv-miR-17-29; ovhv2-miR-17-10 and ovhv2-miR-17-1 respectively[27]. The identification of host targets of the 35 OvHV2-miRNAs is not straightforward. Bioinformatic analysis of potential targets for the OvHV2-miRNAs in the sheep and cattle genome resulted in the identification of more than 100,000 possible targets in each genome, an unrealistic number to investigate. We are current using experimental approaches to investigate cellular targets of the OvHV-2-miRNAs.

## Supporting Information

Table S1 **Sequences of specific forward PCR primers and related annealing temperatures.** The sequence of the forward primers and annealing temperatures used to analyse expression of the predicted ovhv2-miRNAs are shown. Those miRNAs which were successfully validated using approach are shown in bold. “Group 1” etc. represent predicted miRNAs which were not shown to be expressed.
(DOCX)

Table S2 **Sequence of miRNA specific Reverse transcription primers and specific 5′ forward primers.** For each of the miRNAs which were not validated using the miScript assay, the sequence of the primers used to prime cDNA synthesis, the specific 5′ forward primers and the sequence of the universal reverse PCR primer are shown. “Group 1” etc. represent predicted miRNAs which were not shown to be expressed.
(DOCX)
