# Supplementary material for: Expression of Ovine Herpesvirus -2 Encoded MicroRNAs in an Immortalised Bovine – Cell Line
Source: PLoS One. 2014 May 21;9(5):e97765. doi: 10.1371/journal.pone.0097765 (PMC4029829; doi:10.1371/journal.pone.0097765)
Supplement: Table S1 — Sequences of specific forward PCR primers and related annealing temperatures. The sequence of the forward primers and annealing temperatures used to analyse expression of the predicted ovhv2-miRNAs are shown. Those miRNAs which were successfully validated using approach are shown in bold. “Group 1” etc. represent predicted miRNAs which were not shown to be expressed. (DOCX) [file pone.0097765.s001.docx]

**Supplemental Table 1**

**Sequences of specific forward PCR primers and related annealing temperatures.**

| **miRNA** | **Primer sequence** | **RT-PCR Annealing temperature (°C)** |
| --- | --- | --- |
| **Ov2-1** | ATGCTTGTTTAGGCCC | 48 |
| **17-30** | TTTGGGTGTCTCCTG | 46 |
| **17-28** | TCTAGGTTGCATTTTG | 44 |
| **17-27** | CCCACATTTAAGGTG | 44 |
| **17-26** | ATATTCGTTTAGACG | 40 |
| **17-25** | CAATGCTGCTTTGGTG | 48 |
| **17-24** | GGGTTCCTCGAGTGG | 52 |
| **17-23** | ATACACACTGAAAGAGC | 48 |
| **17-22** | ATAAGGCCAACACTAG | 46 |
| **17-21** | AAGCACCTTGGGTGATGTC | 58 |
| 17-19 | AAGCATAGCTGGGAGTG | 52 |
| **17-18** | TAGTAGTCCGTTAACG | 46 |
| **17-16** | TAAACTGGTGGTAGG | 44 |
| **17-15** | TAGCAGTTATGCAGGTATC | 54 |
| **17-14** | TGGCATTTCCAGGAGCCTG | 60 |
| **17-13** | TTGGGTCCAACATGAGA | 50 |
| 17-12 | TATGTCAGAAGTGAAGC | 48 |
| **17-11** | TGGTTTGCATCTGCAC | 52 |
| **17-9** | TAGAGTTACTAAGGATTC | 48 |
| **17-8** | AATCGCCGGTGGCCTTC | 56 |
| 17-7 | TATAGACGGGTATGC | 44 |
| **17-5** | CCTTTTTGGTGAGTTGC | 50 |
| **17-4** | GATTTGATAAAGCCTGC | 48 |
| **17-2** | ACCCCGGGGGTATGTG | 54 |
| **24-1** | GAGCAGTACTACACAGCAG | 58 |
| 61-1 | TTGGGGACGTGCTGGCTG | 60 |
| Group 1 | TCCCGAGATGTCGGG | 50 |
| Group 3 | TTGCGGGGAAGGCCGC | 56 |
| Group 13 | GATGTAGGACAGGCCGC | 56 |
| Group 95 | GCTGCGCTCGCTTGGG | 56 |
| Group 128 | ACGGGTGAGGTGGGGC | 56 |
| Group 181 | CATTGGGGAGGCCGG | 52 |
| Group 182 | AGTGGATGTAGTCCTGG | 52 |
| Group 3p | TCCCGAGATGTCGGG | 50 |
| Group 64p | GGGCGGCTTAGTAAC | 48 |
| Group 96p | TTGATAGCAGGATGTGC | 50 |

The sequence of the forward primers and annealing temperatures used to analyse expression of the predicted ovhv2-miRNAs are shown. Those miRNAs which were successfully validated using approach are shown in bold.

“Group 1” etc. represent predicted miRNAs which were not shown to be expressed.
