# Supplementary material for: Expression of Ovine Herpesvirus -2 Encoded MicroRNAs in an Immortalised Bovine – Cell Line
Source: PLoS One. 2014 May 21;9(5):e97765. doi: 10.1371/journal.pone.0097765 (PMC4029829; doi:10.1371/journal.pone.0097765)
Supplement: Table S2 — Sequence of miRNA specific Reverse transcription primers and specific 5′ forward primers. For each of the miRNAs which were not validated using the miScript assay, the sequence of the primers used to prime cDNA synthesis, the specific 5′ forward primers and the sequence of the universal reverse PCR primer are shown. “Group 1” etc. represent predicted miRNAs which were not shown to be expressed. (DOCX) [file pone.0097765.s002.docx]

**Supplemental Table 2**

**The sequence of miRNA specific Reverse transcription primers and specific 5’ forward primers**

| **miRNA** | **PCR Forward Primer Sequence** | **Reverse Transcription primer sequence** |
| --- | --- | --- |
| 17-19 | GCGGCGAAGCATAGCTGGGAGTG | GTCGTATCCAGTGCAGGGTCCCGAGGTATTCGCACTGGATACGACTCTAGA |
| 17-12 | GCGGCGTATGTCAGAAGTGAAG | GTCGTATCCAGTGCAGGGTCCCGAGGTATTCGCACTGGATACGACTCTCAG |
| 17-7 | GCGGCGGTATAGACGGGTATG | GTCGTATCCAGTGCAGGGTCCCGAGGTATTCGCACTGGATACGACCGGCAG |
| 61-1 | GCGGCTTGGGGACGTGCTGGCTGA | GTCGTATCCAGTGCAGGGTCCCGAGGTATTCGCACTGGATACGACACGTCG |
| 73-1 | GCGGCGTAATCTCTGCTCCAATT | GTCGTATCCAGTGCAGGGTCCCGAGGTATTCGCACTGGATACGACATTTAC |
| Group 1 | GCGGCGGTCCCGAGATGTCGG | GTCGTATCCAGTGCAGGGTCCCGAGGTATTCGCACTGGATACGACCCAGTC |
| Group 3 | GCGGCGTTGCGGGGAAGGCCGC | GTCGTATCCAGTGCAGGGTCCCGAGGTATTCGCACTGGATACGACAAACAC |
| Group 13 | GCGGCGGATGTAGGACAGGCCG | GTCGTATCCAGTGCAGGGTCCCGAGGTATTCGCACTGGATACGACCATCAG |
| Group 95 | GCGGCGCTGCGCTCGCTTGGGGCC | GTCGTATCCAGTGCAGGGTCCCGAGGTATTCGCACTGGATACGACTTGAGG |
| Group 128 | GCGGCGGACGGGTGAGGTGGG | GTCGTATCCAGTGCAGGGTCCCGAGGTATTCGCACTGGATACGACCCCTGC |
| Group 181 | GCGGCGCATTGGGGAGGCCGG | GTCGTATCCAGTGCAGGGTCCCGAGGTATTCGCACTGGATACGACCTTCCC |
| Group 182 | GCGGCAGTGGATGTAGTCCTGGAAG | GTCGTATCCAGTGCAGGGTCCCGAGGTATTCGCACTGGATACGACTAAAGC |
| Group 3p | GCGGCGGTCCCGAGATGTCGG | GTCGTATCCAGTGCAGGGTCCCGAGGTATTCGCACTGGATACGACCCAGTC |
| Group 64p | GCGGCGGGGCGGCTTAGTAA | GTCGTATCCAGTGCAGGGTCCCGAGGTATTCGCACTGGATACGACCACATG |
| Group 96p | GCGGCGTTGATAGCAGGATGTG | GTCGTATCCAGTGCAGGGTCCCGAGGTATTCGCACTGGATACGACCAGGCA |
|  | **PCR Universal Reverse Primer** |  |
|  | GTGCAGGGTCCGAGGT |  |

For each of the miRNAs which were not validated using the miScript assay , the sequence of the primers used to prime cDNA synthesis,the specific 5’ forward primers and the sequence of the universal reverse PCR primer are shown .

“Group 1” etc. represent predicted miRNAs which were not shown to be expressed.
